# Supplementary material for: Episomal and integrated hepatitis B transcriptome mapping uncovers heterogeneity with the potential for drug-resistance
Source: Nat Commun. 2025 Sep 26;16:8515. doi: 10.1038/s41467-025-63497-w (PMC12474897; doi:10.1038/s41467-025-63497-w)
Supplement: Supplementary file 1 — Supplementary Information [file 41467_2025_63497_MOESM1_ESM.pdf]

Episomal and integrated hepatitis B transcriptome mapping uncovers heterogeneity with potential for drug resistance.

Supplementary Material

Harris, JM., Lok, J., Wand, N., Magri, A., Tsukuda, S., Wu, Y., Ng, E., Jennings, D., Elshenwy, B., Balfe, P., and McKeating, JA.

A

Clinical Characteristics	HBeAg+	HBeAg-
	n = 7	n = 39
Sex (female/male)	3/4	13/26
Age (years)	32 (23 - 60)	49 (27 - 86)
Genotype (A/B/C/D/E/Unknown)	0/3/2/1/1/0	3/1/1/24/9/1
Peripheral viral load (log_{10}IU/ml)	7.07 (<1 - 8.13)	3 (<1 - 6.13)
Peripheral HBsAg (log_{10}IU/ml)	4.12 (-2 - 4.43)	3.32 (-0.89 - 4.56)
Ishak liver fibrosis score (0/1/2/3/4/5/6/-)	2/2/2/0/1/0/0/0	5/10/5/4/1/4/1/9
Ishak liver necro-inflammatory score (0-3/4-6/7-9/10+/-)	6/0/1/0/-	20/9/1/1/9

B

Sample Barcode ID	1030	1031	1032	1035	1036	1037	1038	1039	1044	1047	1048
Sex	M	F	M	M	F	F	F	M	M	F	F
Age	59	66	44	49	62	77	58	38	34	63	51
Peripheral viral load (log_{10}IU/ml)	0.95	3.81	3.60	1.18	2.30	3.84	5.68	3.92	0.90	5.20	2.57
Peripheral HBsAg (log_{10}IU/ml)	3.26	3.00	3.92	4.28	1.84	3.08	3.41	3.32	3.67	2.93	2.34
Hepatic cccDNA Positivity (Yes/No)*	Yes	No	Yes	No	No	No	Yes	No	No	Yes	Yes
Hepatic HBs Positivity (0-3)†	1	2	2	1	0	2	1	2	2	3	2
Hepatic HBc Positivity (0-3)†	3	0	2	0	0	0	2	0	0	1	0
Ishak liver fibrosis score (0-6)	6	2	5	3	6	0	5	1	4	3	1
Ishak liver necro-inflammatory score (0-18)	7	2	3	2	3	0	5	1	4	2	0

*cccDNA was detected by ddPCR, and scored as detectable (Yes) or not detectable (No), as previously reported[27].

† Hepatic antigen expression was detected by IHC, with positivity scored on the abundance of antigen expressing cells, see Supplementary Figure 1C

C

HBc Antigen Positivity

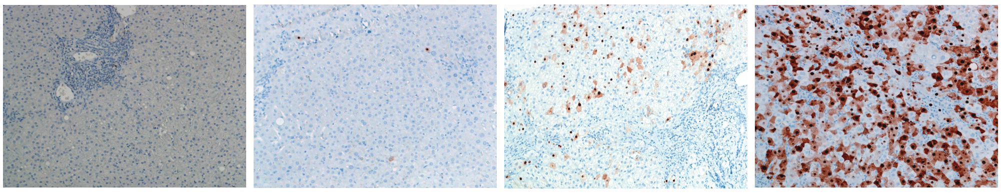

Score:	0	1	2	3
HBc+:	(0%)	(0-33%)	(33-66%)	(66-100%)

Supplementary Figure 1: Clinical characteristics of the patient cohort.

(A) A table of the clinical characteristics of 46 CHB patients, separated by HBeAg positivity. 40 patients were treatment naïve, with 2 HBeAg+ and 2 HBeAg- on antiviral treatment at time of biopsy. Where continuous variables are shown, data are shown as median and range. **(B)** Clinical characteristics of patients selected for further study and immunohistochemical scoring of HBs[27] and HBc antigen stains in the biopsies. **(C)** Representative images of anti-HBc stained biopsies and their assigned score.

A

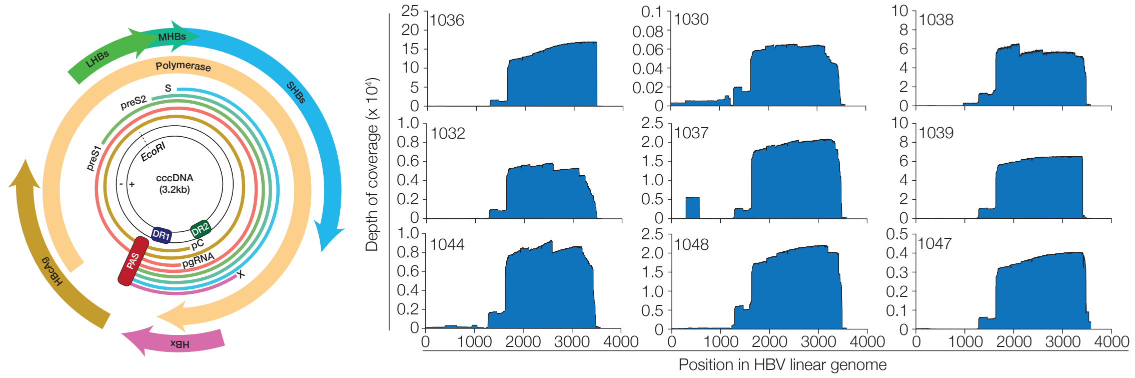

C

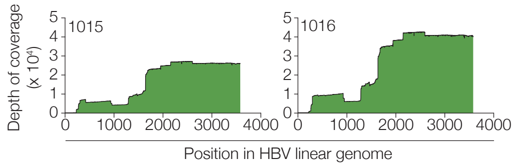

D

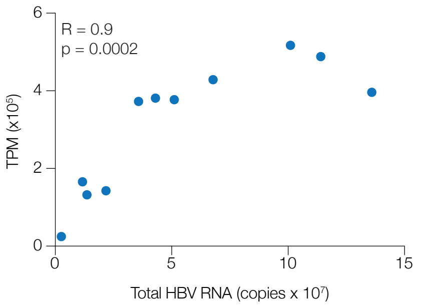

E

Promoter	Altinel et al. TSS	Altinel et al. Range	Harris et al. TSS	Harris et al. Range
Core	pC-L	<13	pC-L	<12
			C-AT1	13-211
	pC-S	229-279	pC-S	212-279
	pgRNA	296±12	pgRNA	296±12
			C-AT2	280-1,196
Sp1	preS1	1290±12	preS1	1,290±12
			Sp1-AT	1,197-1,572*
Sp2	preS2	1628±12	preS2	1,573-1,640
	S	1684±12	S	1,641-1,697
	S(Alt4)	1791±12	Sp2-AT	1,698-2,405
	S(Alt5)	1865±12		
	S(Alt6)	1919±12		
	S(Alt7)	1943±12		
	S(Alt8)	1964±12		
X	X(Alt9)	2418±12	X	2,406-2,430
	X2(Alt13)	3232±12	X-AT	2,431-3,581
Unassigned	pC-L-pC-S	13-228		
	pC-S-pgRNA	279-284		
	pgRNA-preS1	309-1277		
	preS1-preS2	1302-1615		
	preS2-preS	1641-1673		
	S-Alt4	1697-1779		
	S(Alt4)-S (Alt5)	1804-1853		
	S(Alt5)-S (Alt6)	1878-1906		
	S(Alt8)-X(Alt9)	1977-2407		
	X(Alt9)-X2(Alt13)	2431-3220		
	X2(Alt13)-PAS	3244-3581		

Supplementary Figure 2: Long-read sequence coverage and HBV Transcript Start Sites.
(A) A schematic diagram showing the overlapping HBV RNAs (lines), the protein coding ORFs (arrows) and their direction in the circular genome, with key genomic features including the EcoRI site annotated. **(B-C)** Viral reads from nine biopsy samples (blue) and two experimental infections (green) were mapped against an overlength referent genome and the depth of coverage plotted at each nucleotide position. **(D)** The association between the abundance of total HBV RNA as determined by qRT-PCR versus long-read sequencing (TPM) was assessed by Spearman's Rank correlation coefficient. **(E)** Summary of promoter assignment of HBV RNAs along with their transcript start sites (TSS) as previously reported[19]. Viral transcripts were assigned to their cognate promoter based on their TSS and names listed. The TSS range of Sp1-AT is annotated with an * as this flanks the preS1 canonical TSS.

A

Promoter	TSS	TPM (Median & Range) CHB	*In vitro*
Core	pC-L	15 (0-1,251)	1,956 (1,523-2,381)
	C-AT1	0 (0-550)	685 (538-973)
	pC-S	253 (0-8,920)	49,196 (48,493-80,993)
	pgRNA	362 (19-85,393)	16,021 (8,833-31,559)
	C-AT2	1,851 (106-27,553)	22,833 (15,055-23,278)
Sp1	preS1	27,044 (2,651-72,825)	67,735 (45,536-95,018)
	Sp1-AT	4,344 (334-26,625)	57,482 (41,247-59,254)
Sp2	preS2	167,999 (10,289-296,134)	219,509 (176,524-221,624)
	S	38,114 (2,367-79,496)	53,521 (41,939-72,816)
	Sp2-AT	39,922 (2,034-54,329)	33,659 (29,547-35,487)
X	X	1,137 (61-3,981)	448 (408-452)
	X-AT1	17,773 (1,123-42,243)	5,771 (5,748-6,360)

C

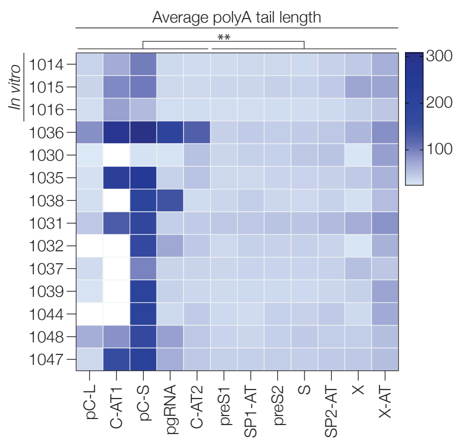

B

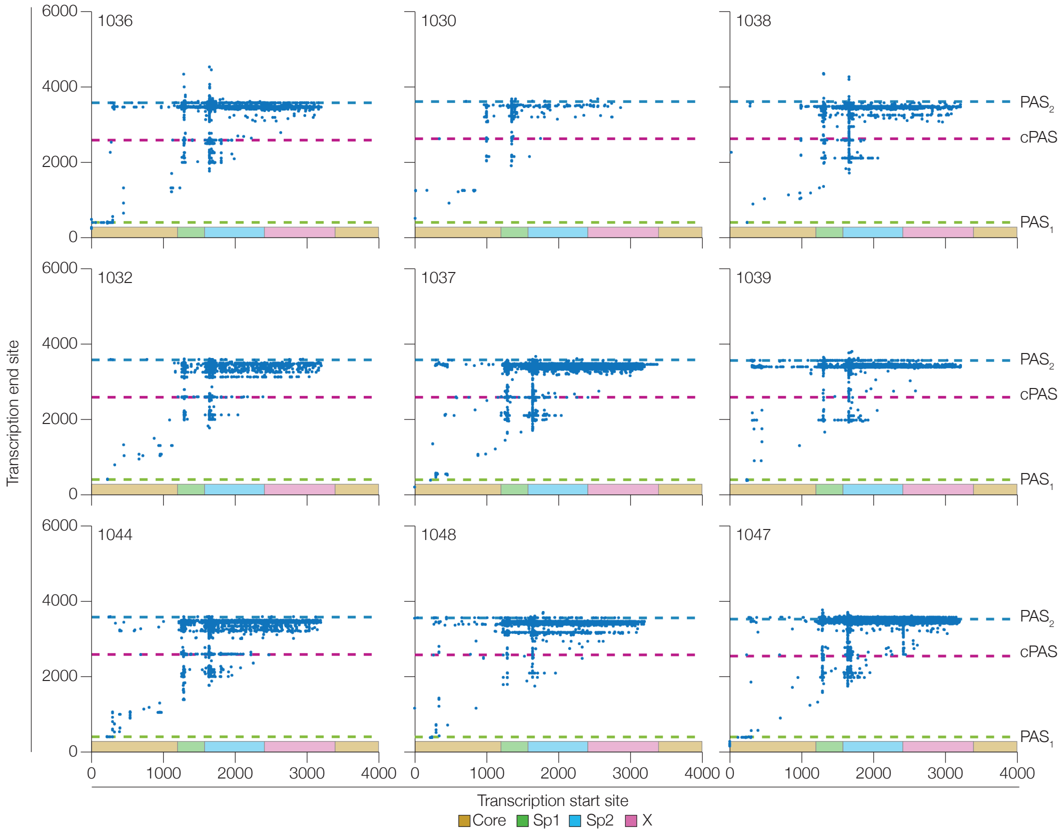

Supplementary Figure 3: HBV Transcript End Sites

(A) Tabulated data showing the abundance of HBV RNAs in 11 CHB patients and 3 in vitro infection samples. Data are listed as mean and range, with transcripts grouped by their respective promoter. (B) Scatter plots showing the Transcription Start Site (TSS) and Transcript End Site (TES) for the HBV RNAs where the position of the PAS1 (400), cPAS (2,600) and the PAS2 (3,581) are shown as dashed lines. Promoter associated regions are depicted as shaded regions on the x axis. (C) The 3' terminus of the HBV RNAs were analysed for polyadenylation and the average length shown on a transcript-by-transcript basis. Average polyA tail length of Core promoter derived transcripts was compared with other viral RNAs in the clinical samples using a Mann-Whitney U test (p=0.0019 **; two-tailed).

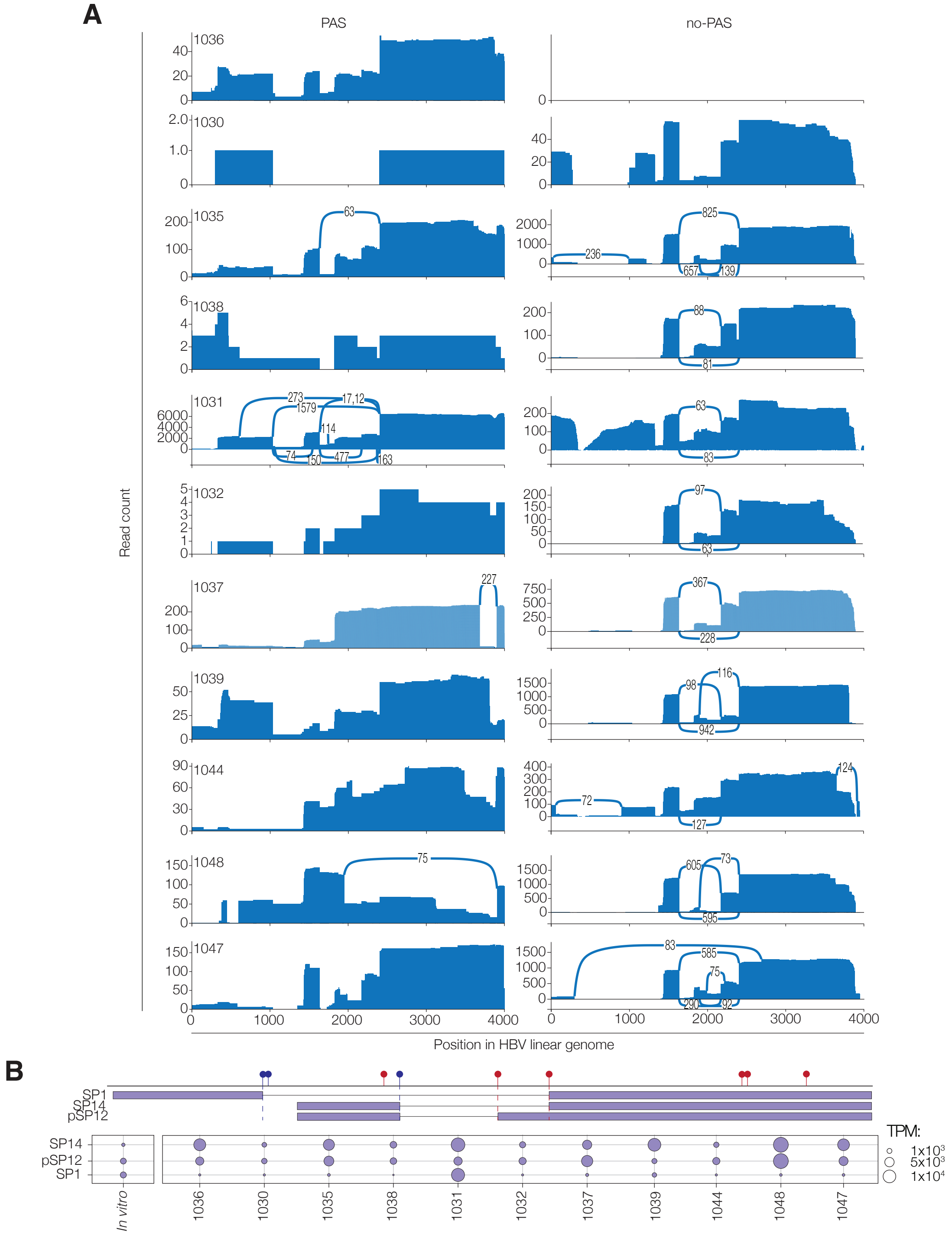

Supplementary Figure 4: Spliced transcript donor:acceptor sites in ccc- and id- RNAs
(A) Transcripts were categorised as PAS or no PAS containing, and for the presence of splice junctions within the HBV encoding fragment. Sashimi plots were generated for the spliced transcripts that derive from cccDNA or iDNA, based on PAS usage. Looping depicts the major splice junction usage in 11 CHB samples. **(B)** A schematic diagram depicting the HBV genome, with splice donor and acceptor sites depicted as blue and red indicators respectively. The 3 most abundant spliced variants are shown, with bubble plots representing their relative abundance, as TPM, in 11 CHB and 1 in vitro (1014) infection samples.

A

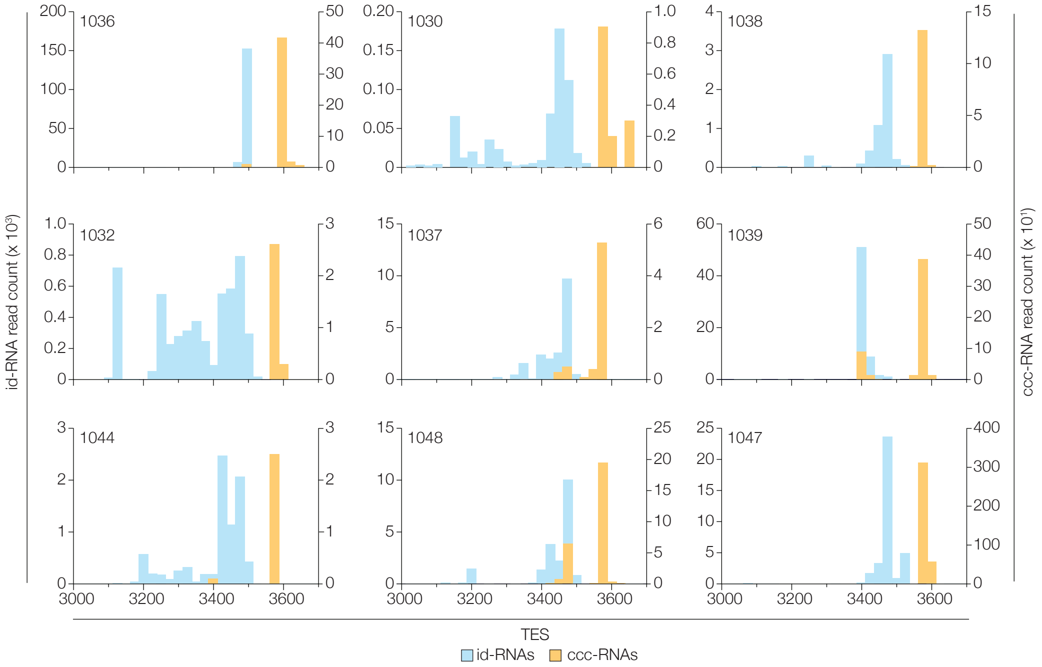

B

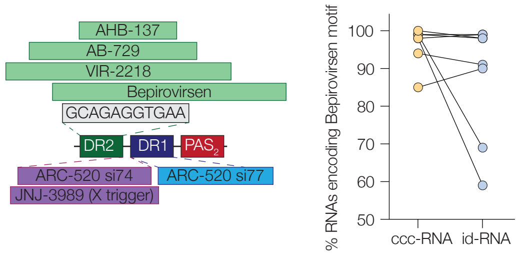

Supplementary Figure 5: Analysing the 3' terminus of HBV RNAs

(A) Histograms showing the termination end site (TES) of id- and ccc- RNAs. The terminal base of each id- and ccc-RNA was assessed, with frequency distribution plotted in 25bp bins from position 3000-4100. id-RNA count is plotted on the left y axis, with bars shown in blue, and ccc-RNAs are depicted by orange bars on the right y axis. (B) A schematic showing the target regions of ASO/siRNA therapeutic agents in the DR2-DR1 loci that have been used in clinical trials. ccc- and id- RNAs were scanned for the presence of the GCAGAGGTGAA motif recognised by Bepirovirsen. If the motif was absent, or had 2 or more mis-matches, it is annotated as not encoding the Bepirovirsen motif. The proportion of ccc- and id- RNAs that encoded the motif are expressed as percentage of reads. Five of the eleven samples were excluded due to low ccc-RNA abundance.

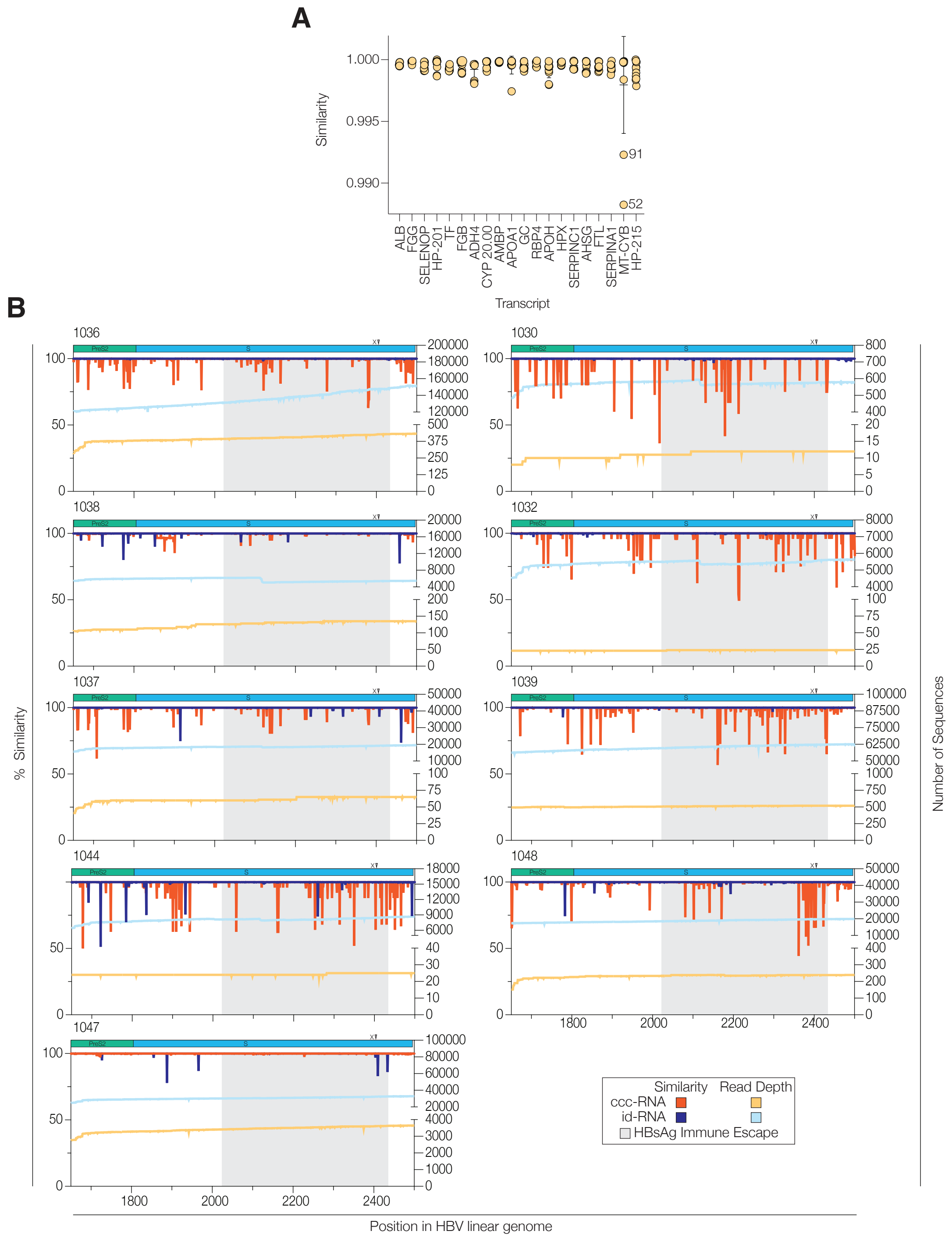

Supplementary Figure 6: Transcript polymorphism in ccc- and integrant derived RNAs
(A) Polymorphism in the 20 most abundant host genes were determined showing greater than 99.8% similarity in all patients, except for MT-CYB transcripts in patient 1048 and 1039, where depth of coverage of these transcripts was very low (52 and 91 transcripts, respectively). **(B)** Waterfall plots showing HBV transcript read depth and similarity against a consensus for cccDNA and iDNA derived RNAs. The grey shading denotes the region that has been previously reported to associate with HBs immune escape mutations.

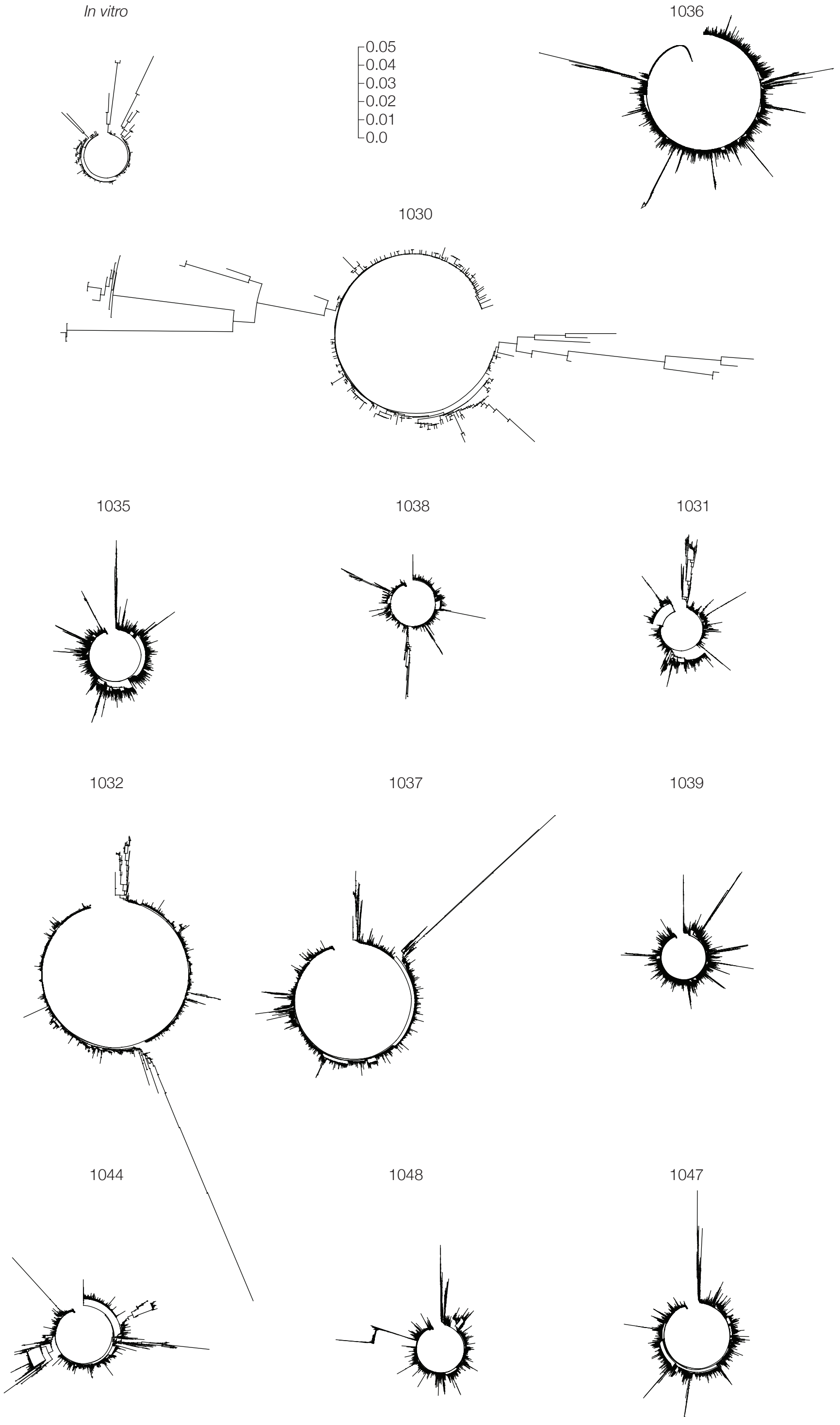

Supplementary Figure 7: Phylogenetic trees of HBV iDNA derived transcripts
HBV reads were assigned as iDNA derived based on the lack of PAS usage. These sequences were analysed using iqtree and phylogenetic trees constructed under the SYM+R3 model. Clades were identified using IQ-tree.

Supplementary Information

Supplementary Table 1: HBV Read counts
Summary of sequencing library composition of each sample. Information is provided on the sequencing barcode, as well as the sample ID. Read counts are tabulated based on the abundance of transcripts that are assigned to each of the previously reported TSS[19], separated as canonical and spliced transcripts.

Supplementary Table 2: Chimeric fusion loci
A table detailing the host genomic locus of each of the integrant derived virus-host chimeras. The Ensemble ID for each of the loci is defined using the Refseq prefixes as follows: NC_ Chromosome; NT_, NW_ contigs or scaffolds, NG_ genes; NM_ coding transcripts; NR_ non-coding; NP_ protein. Each of their frequencies is assessed and annotated.
