# Supplementary material for: Episomal and integrated hepatitis B transcriptome mapping uncovers heterogeneity with the potential for drug-resistance
Source: Nat Commun. 2025 Sep 26;16:8515. doi: 10.1038/s41467-025-63497-w (PMC12474897; doi:10.1038/s41467-025-63497-w)
Supplement: Supplementary file 3 — Description of Additional Supplementary Files [file 41467_2025_63497_MOESM3_ESM.pdf]

Description of Additional Supplementary Files

File Name: Supplementary Data 1
Description: **HBV Read counts**
Summary of sequencing library composition of each sample. Information is provided on the sequencing barcode, as well as the sample ID. Read counts are tabulated based on the abundance of transcripts that are assigned to each of the previously reported TSS19, separated as canonical and spliced transcripts.

File Name: Supplementary Data 2
Description: **Chimeric fusion loci**
A table detailing the host genomic locus of each of the integrant derived virus-host chimeras. The Ensemble ID for each of the loci is defined using the Refseq prefixes as follows: NC_ Chromosome; NT_, NW_ contigs or scaffolds, NG_ genes; NM_ coding transcripts; NR_ non-coding; NP_ protein. Each of their frequencies is assessed and annotated.
