# Supplementary material for: Episomal and integrated hepatitis B transcriptome mapping uncovers heterogeneity with the potential for drug-resistance
Source: Nat Commun. 2025 Sep 26;16:8515. doi: 10.1038/s41467-025-63497-w (PMC12474897; doi:10.1038/s41467-025-63497-w)
Supplement: Supplementary file 6 — Reporting Summary [file 41467_2025_63497_MOESM6_ESM.pdf]

nature portfolio

Corresponding author(s): Jane A McKeating

Last updated by author(s): Aug 19, 2025

Reporting Summary

Nature Portfolio wishes to improve the reproducibility of the work that we publish. This form provides structure for consistency and transparency in reporting. For further information on Nature Portfolio policies, see our Editorial Policies and the Editorial Policy Checklist.

Statistics

For all statistical analyses, confirm that the following items are present in the figure legend, table legend, main text, or Methods section.

n/a	Confirmed	
☐	☒	The exact sample size (*n*) for each experimental group/condition, given as a discrete number and unit of measurement
☐	☒	A statement on whether measurements were taken from distinct samples or whether the same sample was measured repeatedly
☐	☒	The statistical test(s) used AND whether they are one- or two-sided<br>*Only common tests should be described solely by name; describe more complex techniques in the Methods section.*
☒	☐	A description of all covariates tested
☒	☐	A description of any assumptions or corrections, such as tests of normality and adjustment for multiple comparisons
☒	☐	A full description of the statistical parameters including central tendency (e.g. means) or other basic estimates (e.g. regression coefficient) AND variation (e.g. standard deviation) or associated estimates of uncertainty (e.g. confidence intervals)
☐	☒	For null hypothesis testing, the test statistic (e.g. *F*, *t*, *r*) with confidence intervals, effect sizes, degrees of freedom and *P* value noted<br>*Give P values as exact values whenever suitable.*
☒	☐	For Bayesian analysis, information on the choice of priors and Markov chain Monte Carlo settings
☒	☐	For hierarchical and complex designs, identification of the appropriate level for tests and full reporting of outcomes
☒	☐	Estimates of effect sizes (e.g. Cohen's *d*, Pearson's *r*), indicating how they were calculated

Our web collection on statistics for biologists contains articles on many of the points above.

Software and code

Policy information about availability of computer code

Data collection	All sequences were collected on a PacBio Sequel II analyser
Data analysis	All code used in this study is provided as supplementary information as part of the original submission, see file 'Supplementary-Code.pdf'. All packages required for the running of the code are open source (minimap2, samtools and R).

For manuscripts utilizing custom algorithms or software that are central to the research but not yet described in published literature, software must be made available to editors and reviewers. We strongly encourage code deposition in a community repository (e.g. GitHub). See the Nature Portfolio guidelines for submitting code & software for further information.

Data

Policy information about availability of data

All manuscripts must include a data availability statement. This statement should provide the following information, where applicable:

- Accession codes, unique identifiers, or web links for publicly available datasets
- A description of any restrictions on data availability
- For clinical datasets or third party data, please ensure that the statement adheres to our policy

Data availability statement as given in the manuscript:
All data presented in the main and supplementary figures are provided as a Source Data file. The sequencing data generated in this study have been deposited in the NCBI database under accession codes PRJNA1225648 and (https://www.ncbi.nlm.nih.gov/bioproject/PRJNA1225648) and PRJNA1227884 (https://

www.ncbi.nlm.nih.gov/bioproject/PRJNA1227884).
All original code is deposited on Zenodo (http://doi.org/10.5281/zenodo.16539674).

Research involving human participants, their data, or biological material

Policy information about studies with human participants or human data. See also policy information about sex, gender (identity/presentation), and sexual orientation and race, ethnicity and racism.

Reporting on sex and gender	Sex-based analysis were not performed in our study. We did not perform any analysis based on biological sex or any other grouped analysis. Instead, each sample was considered as an individual, and we have reported patient specific observations, noting patterns and trends that appeared across our sample set.<br>We do, however, provide the sex of each of the 11 patients for which material was available for sequencing - these are listed in supplementary table 1.
Reporting on race, ethnicity, or other socially relevant groupings	Neither socially constructed nor socially relevant categorization variables were used in this study. As we considered each sample as an individual, we do not believe that any of the variables between our patients are confounding our analysis.
Population characteristics	N/A: See above.
Recruitment	N/A: Subjects were selected for sequencing based on their disease stage, estimated viral RNA levels (qPCR), and then the quantity and integrity of nucleic acid as determinants for the likelihood of sequencing success.
Ethics oversight	KCH Liver Research 530 Biobank, IRAS project 332608; REC reference 23/LO/0708 and Università del Piemonte Orientale, 531 28100 Novara REC reference CE90/19). All patients consented for surplus tissue to be used for research purposes.

Note that full information on the approval of the study protocol must also be provided in the manuscript.

Field-specific reporting

Please select the one below that is the best fit for your research. If you are not sure, read the appropriate sections before making your selection.

☒ Life sciences ☐ Behavioural & social sciences ☐ Ecological, evolutionary & environmental sciences

For a reference copy of the document with all sections, see nature.com/documents/nr-reporting-summary-flat.pdf

Life sciences study design

All studies must disclose on these points even when the disclosure is negative.

Sample size	No sample size calculation was performed. Patients were selected on the basis of being from a subgroup of patients in the same stage of disease where viral replication is considered to be low level. Further technical considerations such as nucleic acid availability and integrity were made to reach the sample size of 11.
Data exclusions	No data were excluded from the study, all findings are reported.
Replication	N/A: It was not possible to have replicate samples from the clinical samples. However, the inclusion of n=3 independent biological replicates from the in vitro infection samples show the reproducibility of the data.
Randomization	N/A: This is not relevant to our study, each sample was considered as a patient specific observation. Where comparisons were made (Figure 1) diagnostic disease classifications were used to determine the grouping, as detailed in supplementary figure 1.
Blinding	All samples were anonymized before delivery to the lab. Blinding was not possible for our study, all our samples are from a cohort of patients who were treatment naive, and in the same phase of disease.

Reporting for specific materials, systems and methods

We require information from authors about some types of materials, experimental systems and methods used in many studies. Here, indicate whether each material, system or method listed is relevant to your study. If you are not sure if a list item applies to your research, read the appropriate section before selecting a response.

Materials & experimental systems

n/a	Involved in the study
☒	☐ Antibodies
☐	☒ Eukaryotic cell lines
☒	☐ Palaeontology and archaeology
☒	☐ Animals and other organisms
☐	☒ Clinical data
☒	☐ Dual use research of concern
☒	☐ Plants

Methods

n/a	Involved in the study
☒	☐ ChIP-seq
☒	☐ Flow cytometry
☒	☐ MRI-based neuroimaging

Eukaryotic cell lines

Policy information about cell lines and Sex and Gender in Research

Cell line source(s)	HepG2-NTCP cells, immortalized human hepatoma cell lines that are engineered to overexpress the NTCP receptor. These cells were a gift from Prof Stephan Urban (University of Heidelberg).
Authentication	HepG2 cell line originally from ATCC (product HB-8065).
Mycoplasma contamination	Cells were verified to be Mycoplasma free on receipt from Heidelberg.
Commonly misidentified lines (See ICLAC register)	*Name any commonly misidentified cell lines used in the study and provide a rationale for their use.*

Clinical data

Policy information about clinical studies

All manuscripts should comply with the ICMJEguidelines for publication of clinical research and a completedCONSORT checklist must be included with all submissions.

Clinical trial registration	N/A
Study protocol	N/A
Data collection	N/A
Outcomes	N/A

Plants

Seed stocks	N/A
Novel plant genotypes	N/A
Authentication	N/A
